# Gluten-free Diet Reduces the Risk of Irritable Bowel Syndrome: A Mendelian Randomization Analysis

**DOI:** 10.3389/fgene.2021.684535

**Published:** 2021-11-09

**Authors:** Yuhao Sun, Xuejie Chen, Shuyang Wang, Minzi Deng, Ying Xie, Xiaoyan Wang, Jie Chen, Therese Hesketh

**Affiliations:** ^1^ Centre for Global Health, Zhejiang University School of Medicine, Hangzhou, China; ^2^ Department of Gastroenterology, The Third Xiangya Hospital, Central South University, Changsha, China; ^3^ Institute for Global Health, University College London, London, United Kingdom

**Keywords:** irritable bowel syndrome, gluten-free diet, mendelian randomization, causal association, genome-wide association studies

## Abstract

**Background**: Whether a gluten-free diet (GFD) is a cause of irritable bowel syndrome (IBS) remains controversial. We aim at exploring the causal relationship between gluten intake and IBS within Mendelian randomization (MR) design.

**Methods:** We conducted a two-sample MR and selected single-nucleotide polymorphisms (SNPs) associated with GFD as instrumental variables (IVs). SNPs and genetic associations with GFD and IBS were obtained from the latest genome-wide association studies (GWAS) in Europeans (GFD: cases: 1,376; controls: 63,573; IBS: cases:1,121; controls: 360,073). We performed inverse variance weighting (IVW) as the primary method with several sensitivity analyses like MR-Egger and MR-PRESSO for quality control. The above analyses were re-run using another large dataset of IBS, as well as changing the *p*-value threshold when screening IVs, to verify the stability of the results.

**Results:** The final estimate indicated significant causal association [per one copy of effect allele predicted log odds ratio (OR) change in GFD intake: OR = 0.97, 95% confidence interval (CI) 0.96 to 0.99, *p* < 0.01] without heterogeneity statistically (Q = 2.48, *p* = 0.78) nor horizontal pleiotropy biasing the causality (*p* = 0.92). Consistent results were found in validation analyses. Results of MR Steiger directionality test indicated the accuracy of our estimate of the causal direction (Steiger *p* < 0.001).

**Conclusion:** GFD might be a protective factor of IBS. Therefore, we suggest taking a diet of lower gluten intake into account in IBS prevention and clinical practice.

## Introduction

Irritable bowel syndrome (IBS) is a functional gastrointestinal disorder with recurrent abdominal pain associated with defecation and bowel habit change (Ford et al., 2020). Considered as the most common chronic functional gastrointestinal disease, it affects 5–10% of the world’s population, significantly deteriorating the quality of life (El-Salhy, 2015; Rej & Sanders, 2018; Flacco et al., 2019; Oka et al., 2020).

Since diet was assumed to be included in the pathophysiology of IBS and food ingestion triggered symptoms among up to 60% of IBS patients, controlling diets might be a primary prevention approach with low cost and high yield. Dietary modification like a gluten-free diet (GFD) was recommended to relieve their symptoms because of the overlap between IBS and gluten sensitivity (Lahner et al., 2013; De Giorgio et al., 2016); Verdu et al., 2009; Biesiekierski et al., 2011). However, when taking the confounding factors into account, the results of observational studies can be the opposite (Bowden et al., 2017). There is little high-quality evidence exploring the effect of specific diets on IBS (Ford et al., 2020), and whether a causal association between GFD and the development of IBS exists remains unanswered.

To investigate the causality of GFD and the lower risk of incident IBS, we conducted two-sample Mendelian randomization (MR). According to Mendel’s law of inheritance, genetic variants are randomly assigned prior to the onset of disease, and thus genetic variants are improbable to be downstream events of subsequently measured events. Thus, in the MR analysis, we selected appropriate genetic variants [single-nucleotide polymorphisms (SNPs)] as instrumental variables (IVs) for the exposure. In this way, it is possible to avoid reverse causality and confounding factors such as psychological and environmental factors (Saito et al., 2005; Bowden et al., 2017). Additionally, in the two-sample MR study, information on gene–outcome association and gene–exposure association were obtained from two separate samples of the same population. Based on existing data, it is more economical and efficient (Pierce & Burgess, 2013).

## Materials and Methods

### Study Design

**FIGURE 1:**
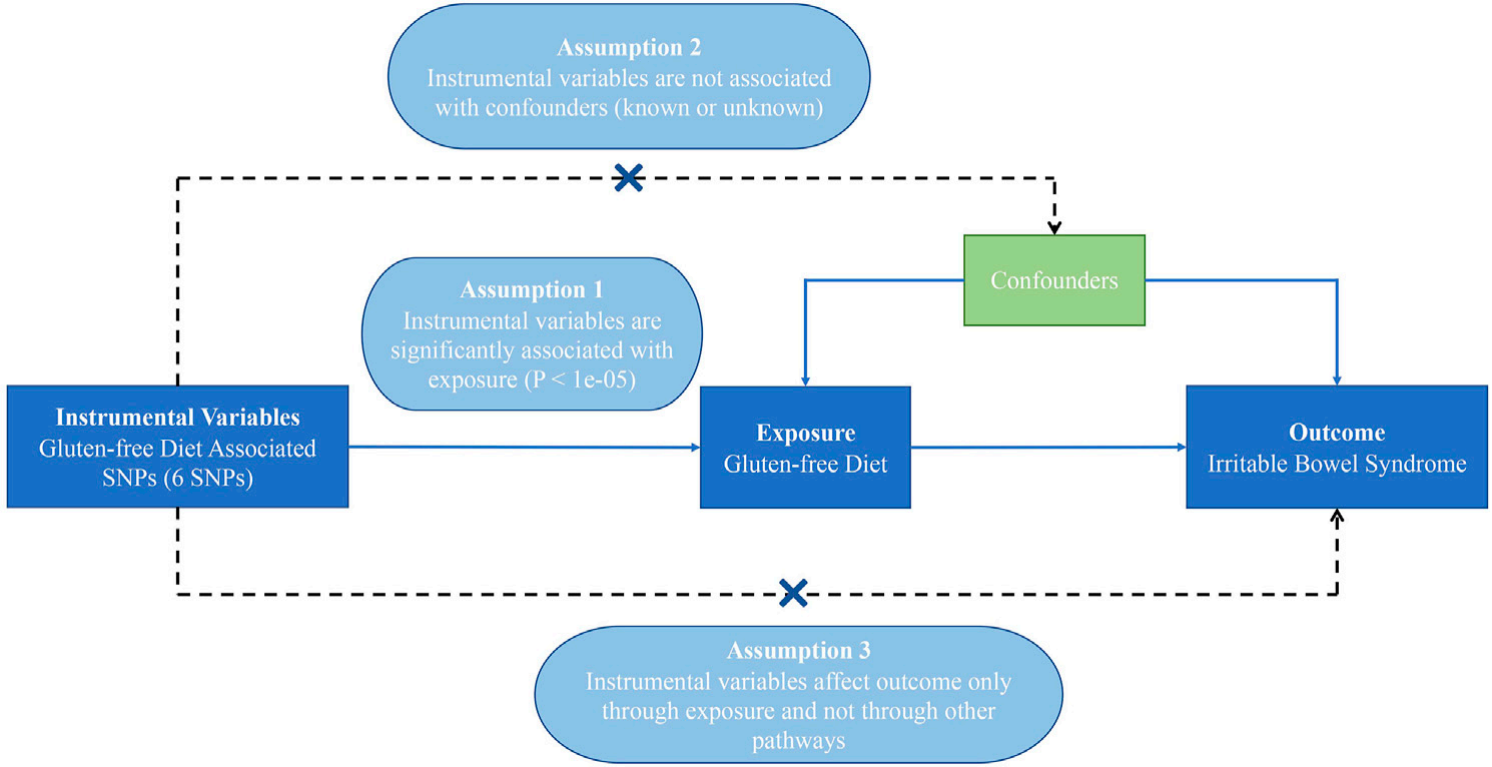
Diagram of the Mendelian randomization assumptions supporting a two-sample Mendelian randomization analysis of the causal effect of gluten-free diet on irritable bowel syndrome (the dashed lines indicate possible causal effects between variables which may be against the Mendelian randomization assumptions).

In this MR analysis, we extracted genetic variants that are strongly associated with GFD from open genome-wide association studies (GWAS) as IVs and used IVs to replace GFD as new exposures to investigate the causal relationship between GFD and IBS. The extracted IVs must meet three core assumptions: 1) be strongly associated with the exposure; 2) be independent of confounders; and 3) influence the outcome exclusively via the exposure (details in Figure 1) (Bowden et al., 2015; Bowden & Holmes, 2019). The ratio of the gene–outcome association and gene–exposure association obtained from GWAS could assess the causal association between exposure and outcome (Lawlor et al., 2008). The total effect was calculated using the inverse variance weighting (IVW) method to summarize the estimate of every single IV. Sensitive analyses were conducted, including MR-Egger and MR-PRESSO to estimate horizontal pleiotropy, maximum likelihood (ML), weighted median (WME), robust adjusted profile score (RAPS), MRCIP, and MR-PRESSO for result validation. Furthermore, considering the small number of IBS cases, we used an outcome dataset with a larger number of cases (10,939) for validation. All the data we used were selected from publicly available databases with ethical approval in origin.

### Data Source

GWAS provided large-sized, repeatedly checked samples, which made our studies available and reliable. We obtained genetic predictors and genetic associations with GFD from the latest and biggest GWAS of GFD (Elsworth et al., 2020). The study ascertained GFD standards with the Oxford WebQ (an online questionnaire reporting the condition of food intaking), and participants who follow a GFD or wheat-free diet routinely were considered as GFD cases. The summary data covered the range of 64,949 European individuals (cases: 1,376, controls: 63,573) (Hemani et al., 2018; Liu et al., 2011).

Genetic associations with IBS were acquired from the latest United Kingdom Biobank GWAS study. United Kingdom Biobank collects the deep genetic and traits data of 488,377 individuals, including 361,194 individuals with IBS genetic information (cases: 1,121, controls: 360,073) (Bycroft et al., 2018). IBS cases refer to participants who have recurrent abnormal pain or discomfort for at least 3 days per month during the last 3 months accompanied by two or more of the following conditions: 1) symptom ease after defecation, 2) changes in defecation frequency during seizures, and 3) seizure with stool changes (Spiller, 2003; Enck et al., 2016). The GWAS used BOLT-LMM106 to adjust for sex and age, and the first 20 ancestor principal components (PCs) as covariates (Wu et al., 2021).

The dataset for validation was a large GWAS based on the UK biobank, 2021 (cases: 10,939, controls: 451,994) (Elsworth et al., 2020). In this study, the outcome was self-reported IBS, obtained from verbal interviews by trained nurses.

### Selection of Instrumental Variables

Appropriate SNPs must be linked to GFD strongly (*p* < 5 × 10^–8^) with low linkage disequilibrium (LD) (*r* ^2^ < 0.001) and high minor allele frequency (MAF > 0.05). A higher genome-wide significance (*p* < 1 × 10^–5^) was also used to obtain more SNPs to predict GFD since only three SNPs were included under the lower threshold (*p* < 5 × 10^–8^). SNPs associated with IBS were excluded, and proxies were available (*r* ^2^ > 0.8) when SNPs were not found in the outcome databases (Machiela & Chanock, 2015). Palindromic SNPs with high MAF (>0.3) are removed to avoid the ambiguous inference of orientation. Meanwhile, F-statistics was calculated to check the bias caused by weak IVs (F-statistics should be at least greater than 10, preferably more than 100) (Bowden et al., 2016b; Burgess et al., 2016).

To reduce the interference from confounders, each instrumental SNP was estimated for the possible connections with confounders including age when full-time education was completed (226,899 individuals), the year full-time education ended (112,569 individuals), circulating leptin levels adjusted for body mass index (BMI) (33,987 individuals), average total household income before tax (397,751 individuals), age of smoking initiation (341,427 individuals), and alcoholic drinks per week (335,394 individuals) (details in Supplementary Figure S1 and Table S2) (Kilpeläinen et al., 2016; Bowden et al., 2017; Liu et al., 2019).

### Statistical Analysis

All statistical analyses were performed in R 4.0.3 with R package “TwoSampleMR” and “MRPRESSO.”

#### Primary Statistical Analysis

IVW was chosen as the primary analysis, as it is the most efficient method to estimate the causal effect when the included IVs are all valid (satisfied the InSIDE assumption) (Bowden et al., 2015) and the intercept of the IVW regression equation is assumed to be 0 (Bowden et al., 2017).

The causal association between exposure and outcome $\left({\hat{\beta }}_{j}\right)$ could be estimated by the ratio of the SNP–outcome association ( ${\hat{\Gamma }}_{j}$ ) and the SNP–exposure association ( ${\hat{\gamma }}_{j}$ ) (Lawlor et al., 2008):

$${\hat{\beta }}_{j}=\frac{{\hat{\;\Gamma }}_{j}}{{\hat{\gamma }}_{j}}.$$

When genetic variants were not related, we estimated an overall effect combining the estimated ratio of each variant, called IVW estimator. IVW estimator refers to a consistent estimate of the causal association if IVs meet all IVs assumptions [ $\sigma_{\mathrm{Yj}}$ refers to the standard error (SE) of the SNP–outcome (Y) estimate of SNP_j_] (Burgess et al., 2013; Johnson, 2014):

$${\hat{\beta }}_{\mathrm{IVW}}=\frac{\sum_{j}{\hat{\gamma }}_{j}^{2}\sigma_{\mathrm{Yj}}^{-2}{\hat{\beta }}_{j}}{\sum_{j}{\hat{\gamma }}_{j}^{2}\sigma_{\mathrm{Yj}}^{-2}}.$$

#### Sensitive Analyses

The MR analysis was also conducted in different methods to check the robustness of the results. Other analysis methods that were exerted included MR Egger, ML, WME, RAPS, MRCIP, and MR-PRESSO. Each method has its own characteristics. MR Egger contains the square of SE as its weight, and the intercept is taken into consideration (Bowden et al., 2017). The probability of each nucleotide substitution is considered in ML aiming to find a phylogenetic tree that can engender observation data in a higher possibility (Luque-Fernandez et al., 2018). WME is robust to outliners focusing on the median of the distribution function estimated by IVW to analyze (Bowden et al., 2016a). RAPS can downweight outliers, while MRCIP is a new robust MR method that can handle correlated and idiosyncratic pleiotropy at the same time through the PRW-EM algorithm combined with expectation–maximization (EM) algorithm (Zhao et al., 2020; Xu et al., 2021). MR-PRESSO was conducted to estimate the horizontal pleiotropy and the total effect after removing outliers (Verbanck et al., 2018).

When conducting MR analysis, the three core assumptions above may be violated due to the horizontal pleiotropy, leading to inaccurate results. Pleiotropy includes vertical and horizontal pleiotropy. Since eliminating the horizontal pleiotropy would make the result more reliable while removing the vertical one might distort the causal estimation, we only test horizontal pleiotropy (Bowden et al., 2017). The test methods were comparing the intercept of the MR Egger function with 0 and applying MR-PRESSO. We also search the GWAS Catalog to avoid more specific pleiotropic paths (Buniello et al., 2019). Besides, the heterogeneity was quantified by the *p*-value derived from Cochran’s Q test and checked deeper by the leave-one-out sensitivity test.

In our design, we assumed that IVs subsequently affect outcomes by influencing exposure. Therefore, we used the MR Steiger directionality test to examine whether the results we found followed the direction in our hypothesis (Hemani et al., 2017). The Steiger *p*-value of less than 0.05 was considered to show the correct direction.

## Result

According to the criteria mentioned above, we extracted three SNPs that met our inclusion criteria (rs1548306, rs9271842, and rs9273595) and three more SNPs under the lower *p*-value threshold (*p* < 1 × 10^–5^) (rs2148682, rs2282910, and rs9277568) as IVs to conduct MR. As rs9271842 was not included in our outcome database; rs9271847 was used as a proxy to gain the gene–outcome association. F-statistics ranging from 20.02 to 122.20 indicated less likely bias caused by weak IVs (details in Table 1; Supplementary Table S1).

**TABLE 1:** Genetic associations of GFD on IBS (at *p* < 1 × 10^–5^).

SNP^a^	Chr	Position	A1/A2	Adjacent gene	Beta	SE	*p*	OR (95%CI)	F^b^
rs1548306	6	32427179	T/A	CREB5	−0.02	0.02	0.27	0.98 (0.94, 1.02)	73.34
rs2148682	1	65869489	C/T	HLA-DRA	−0.03	0.04	0.47	0.97 (0.90, 1.05)	20.02
rs2282910	7	28844824	T/C	HLA-DQB1	0.02	0.04	0.65	1.02 (0.95, 1.10)	20.37
rs9271842	6	32594953	A/C	DNAJC6	−0.05	0.02	0.04	0.95 (0.91, 1.00)	43.49
rs9273595	6	32629091	G/C	HLA-DPB1	−0.02	0.02	0.13	0.98 (0.95, 1.01)	122.20
rs9277568	6	33057055	C/T	HLA-DRB5	−0.04	0.03	0.20	0.96 (0.90, 1.02)	29.26

GFD, gluten-free diet; IBS, irritable bowel syndrome; SNP, single-nucleotide polymorphism; Chr, chromosome; A1, effect allele; A2, other allele; SE, standard error; OR, odds ratio; CI, confidence interval.

a Rs1548306, rs9271842, and rs9273595 were extracted as instrumental SNPs under a smaller *p*-value threshold (*p* < 5 × 10^–8^).

b The F-statistics was calculated by the formula: $\text{F}=\frac{\beta_{SNP-exposure}^{2}}{Variance_{SNP-exposure}}$ .

The results of IVW with three IVs illustrated a suggestive effect of GFD on the risk of IBS [per one copy of effect allele predicted log odds ratio (OR) change in GFD intake: OR = 0.97, 95% confidence interval (CI) 0.95 to 0.99, *p* < 0.05]. A consistent result was found when six IVs were included into the analysis (IVW, OR = 0.97, 95%CI 0.96 to 0.99, *p* < 0.01).

**FIGURE 2:**
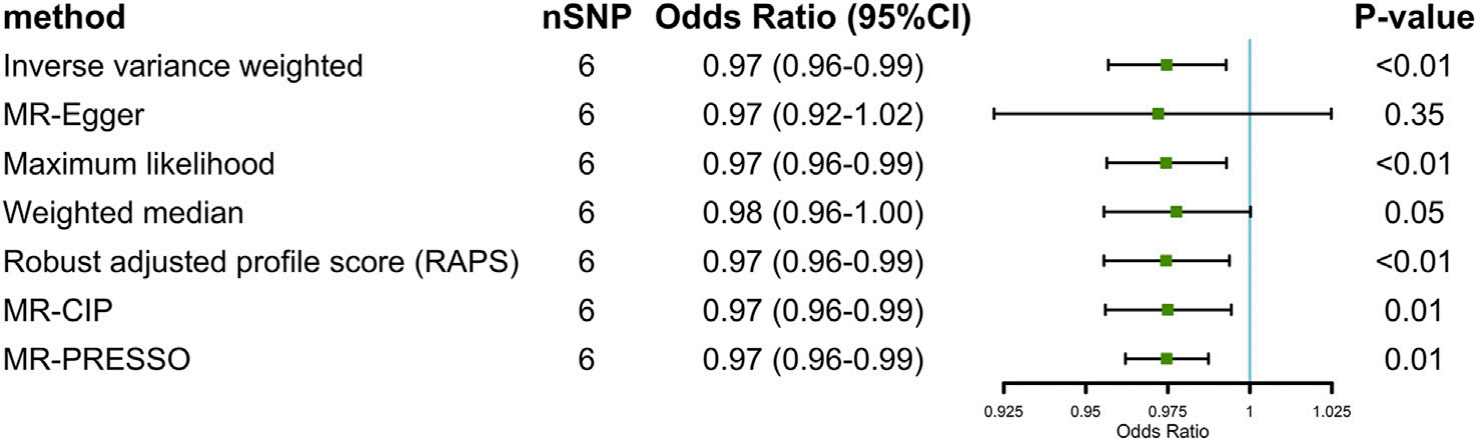
Two-sample Mendelian randomization estimations of the causal effect of gluten-free diet on irritable bowel syndrome with IVW, MR Egger, ML, WME, RAPS, MRCIP, and MR PRESSO (at *p* < 1 × 10^–5^).

Given the insufficient number of three IVs to complete only partial sensitivity analyses, we completed sensitivity analyses primarily when six IVs were available. In sensitive analyses, results in the other six methods (MR Egger, ML, WME, RAPS, MRCIP, and MR-PRESSO) all went along well with what we supposed despite some small deviations. The scatter plot also showed that the curves fitted by the various methods were generally consistent across parameters (details in Figure 2; Supplementary Figure S2). We found no statistically significant horizontal pleiotropy [MR Egger (intercept): *p* = 0.51 (3 IVs), *p* = 0.92 (6 IVs); MR-PRESSO: *p* = 0.85 (6 IVs, three IVs not enough for this method)] or heterogeneity in the IVW method [Q = 0.95, *p* = 0.63 (3 IVs); Q = 2.48, *p* = 0.78 (6 IVs)] (additional funnel plot in Supplementary Figure S3), and no obvious factors were found when searching the GWAS Catalog that could cause possible pleiotropy. Furthermore, we conducted leave-one-out sensitivity estimations by extracting one of the six SNPs out each time. The result of leave-one-out sensitivity estimations illustrated that every SNP of the six hardly biases the outcome (details in Supplementary Figure S4). Results of the MR Steiger directionality test indicated the accuracy of our estimate of the causal direction (Steiger *p* < 0.001).

After changing the outcome dataset for validation, we still observed that GFD was associated with a lower risk of IBS (IVW, OR = 0.93, 95%CI 0.89 to 0.97, *p* < 0.01) without statistically significant horizontal pleiotropy (*p* = 0.51) or heterogeneity (Q = 5.43, *p* = 0.37).

## Discussion

In this study, we found that GFD was associated with a lower risk of IBS, and this causal effect provided evidence for IBS prevention and was valuable for future investigations and clinical practice.

Accumulated previous studies proved that specific food, especially gluten, might play a key role in inducing and worsening some symptoms of IBS (Zar et al., 2001; Atkinson et al., 2004; Spencer et al., 2014). With regard to the putative mechanism, some studies have pointed out that gluten might change the bowel barrier and increase the permeability of the small bowel, evoking inflammation and higher sensitivity (Vazquez-Roque et al., 2013; Shahbazkhani et al., 2015). As the initial stimulator of hypersensitivity, bowel inflammation could cause lasting mild inflammation and bowel irritability, which in turn generate or exacerbate IBS (Neal et al., 1997; Spiller et al., 2000; Gwee et al., 2003). Additionally, epithelial T lymphocytes and mast cells proliferated slightly under the colonic biopsies further verified the role of inflammation in the onset of IBS (Barbara et al., 2004; Ohman & Simrén, 2010). Besides, it was interesting to note that except for bowel reactions, gluten also showed systemic effects including the sense of fatigue (Biesiekierski et al., 2013; Shahbazkhani et al., 2015). Since psychological factors are crucial to the pathogenesis of IBS, the protective effect of GFD on IBS may also be explained in this way (Drossman, 1999).

To our knowledge, this was the first MR study providing insights on the preventive effect of GFD, while the long-term adherence to GFD was inversely associated with IBS development. It is widely acknowledged that strict diet control was hardly a long-term intervention, which makes related randomized control trials (RCT) evidence insufficient. Meanwhile, our MR study could overcome the susceptible bias of confounders and reverse causality shared by traditional observational studies.

Although the current MR study was performed rigorously and had plenty of strengths, some limitations exist. Firstly, a spurious causal relationship may be caused by the possible difference of frequency in people with diverse genetic backgrounds. To minimize this bias, our IVs were all selected from genetic variants from sources of European ancestry and genomic control research. Nevertheless, our results in the European group might not generalize to other races (Garcia-Mazcorro et al., 2018). Secondly, in MR, it was a challenge to eliminate the interferences of potential confounders and horizontal pleiotropy. Therefore, we applied six other methods besides IVW and found consistent results. Besides, MR Egger and MR-PRESSO were employed to assess horizontal pleiotropy and found unlikely to exhibit such bias. Furthermore, before conducting MR analysis, we had estimated associations between some confounders and SNPs and found that the SNPs all met IV assumption two which ensured higher accuracy and reliability (details in Supplementary Table S2). Thirdly, as the self-administered questionnaire of GFD from United Kingdom Biobank only presents binary choices, Yes/No, the effect of GFD on preventing IBS could be only described qualitatively rather than quantitatively. Detailed investigation on gluten intake might show more information, but we were not aware of any available large GWAS related at present. Fourthly, the limited number of cases and coefficient of determination make the statistical power relatively insufficient. To minimize this effect, we verified our hypothesis in another larger dataset. Considering the limited statistical power, larger and higher-quality GWAS studies or well-designed trials should be conducted to validate this conclusion. Last but not least, the health benefit of following a GFD among the population without gastrointestinal diseases like IBS remains a core controversial issue for public health. Further studies are necessary because of the current lack of experimental evidence (Staudacher and Gibson, 2015; Dionne et al., 2018; Melini & Melini, 2019). Thus, we still need to be vigilant about potential dietary change risks and inducing nutrition deficiency while commencing and adhering to a GFD.

Furthermore, our results demonstrated the protective effect of GFD, but GFD is not exactly the same as just reducing gluten intake, especially when there were some other components in the gluten-containing food. Fructans, another key ingredient found in gluten-containing foods demonstrated adverse effects not only on the bowel but also on mental problems. An RCT directly clarified that fructan rather than gluten was regarded as a culprit (Skodje et al., 2018). Several RCTs also compared FODMAPs (low fermentable, oligosaccharides, disaccharides, monosaccharides, and polyols) with GFD and found positive results, since fructan was an oligosaccharide in FODMAPs (Halmos et al., 2014; Dionne et al., 2018; Krieger-Grübel et al., 2020). Therefore, whether gluten was a crucial step in GFD to develop IBS or merely an inducer still needs further investigation on other ingredients in gluten-containing foods.

## Conclusion

Our result provided suggestive evidence of a protective causal effect of GFD on the IBS incident. Therefore, we suggested taking a diet of reducing gluten intake into account in terms of IBS prevention in clinical practice.

## Data Availability

The original contributions presented in the study are included in the article/Supplementary Material, further inquiries can be directed to the corresponding authors.
