# Supplementary material for: Gluten-free Diet Reduces the Risk of Irritable Bowel Syndrome: A Mendelian Randomization Analysis
Source: Front Genet. 2021 Nov 9;12:684535. doi: 10.3389/fgene.2021.684535 (PMC8660079; doi:10.3389/fgene.2021.684535)
Supplement: Supplementary file 1 [file DataSheet1.docx]

Supplementary Materials

Title: Gluten-Free Diet Reduces the Risk of Irritable Bowel Syndrome: A Two-sample Mendelian Randomization Analysis

**Figure S1.** Data Sources and Confounders Estimation Plan via Two-sample Mendelian Randomization.

Figure S2. Scatter Plot of Single-nucleotide Polymorphisms via Two-sample Mendelian Randomization with IVW, MR Egger, ML, WME, and RAPS

Figure S3. Funnel Plot of Single SNPs Used in the Two-sample Mendelian Randomization Analysis with Inverse variance weighted and MR Egger

Figure S4. Leave-One-Out Test for Single SNPs Used in the Two-sample Mendelian Randomization Analysis of the Causal Effect of Gluten-Free Diet on Irritable Bowel Syndrome

Table S1. Detail Information of Instrumental SNPs

Table S2. Associations between Confounders and Single-nucleotide Polymorphisms via Two-sample Mendelian Randomization

**Figure S1.** Data Sources and Confounders Estimation Plan via Two-sample Mendelian Randomization.

^a^ Summary data were obtained from Genome-Wide Association Studies (GWAS). For the sample size of each study, see SNP selection.

^b^ Any SNP which associates with one or more confounders is against the Mendelian randomization IV assumption 2. None of the SNPs was used as IV in our study has pleiotropic associations with con-founders we selected.

IEU - Integrative Epidemiology Unit; GSCAN - Sequencing Consortium of Alcohol and Nicotine use; LD - Linkage disequilibrium.


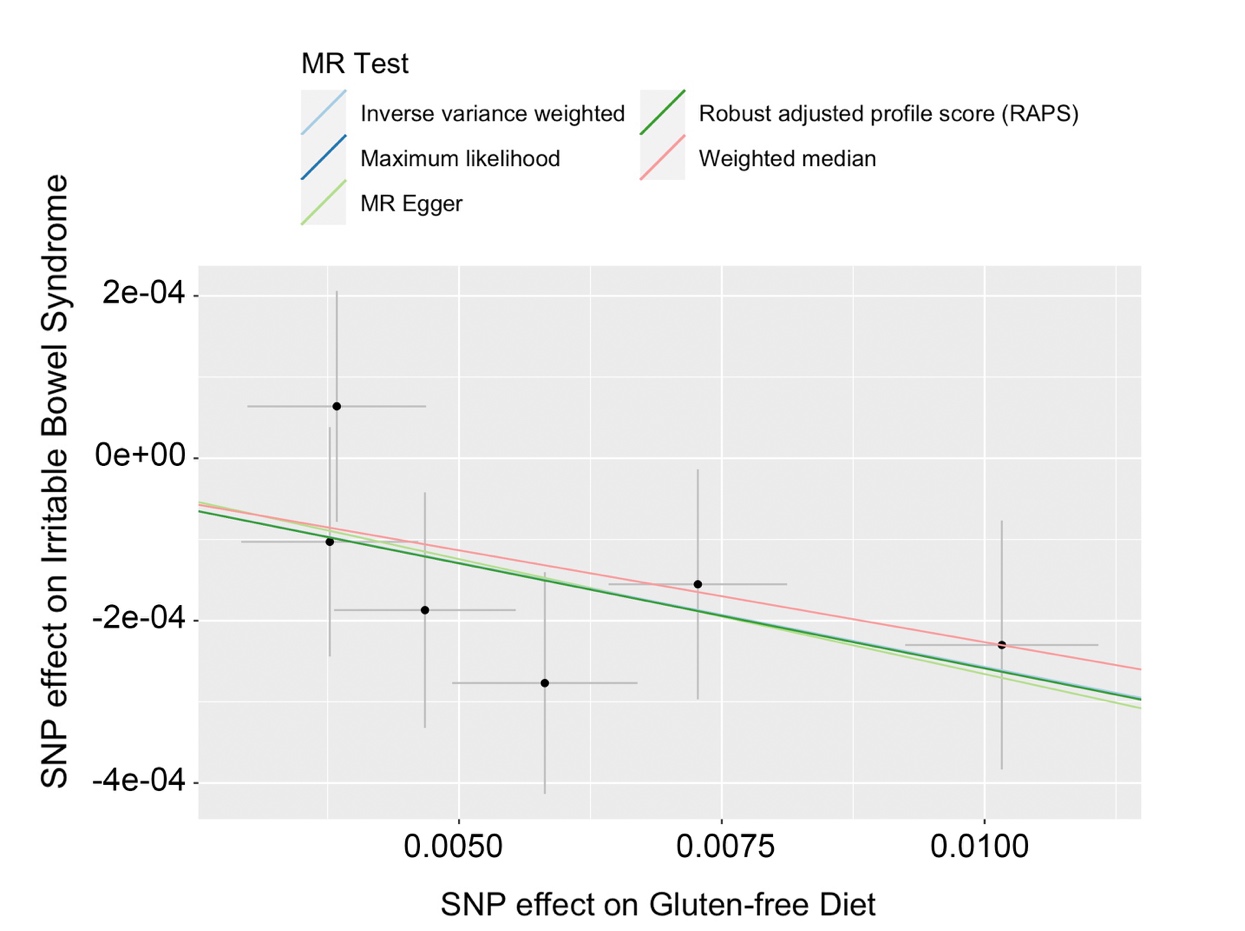


Figure S2. Scatter Plot of Single-nucleotide Polymorphisms via Two-sample Mendelian Randomization with IVW, MR Egger, ML, WME, and RAPS (at *p<1×10^-5^*)

*Abbreviations*: IVW - Inverse variance weighted; ML - Maximum likelihood; WME - Weighted median; SNP - single-nucleotide polymorphism.

Figure S3. Funnel Plot of Single SNPs Used in the Two-sample Mendelian Randomization Analysis with Inverse variance weighted and MR Egger (at *p<1×10^-5^*)


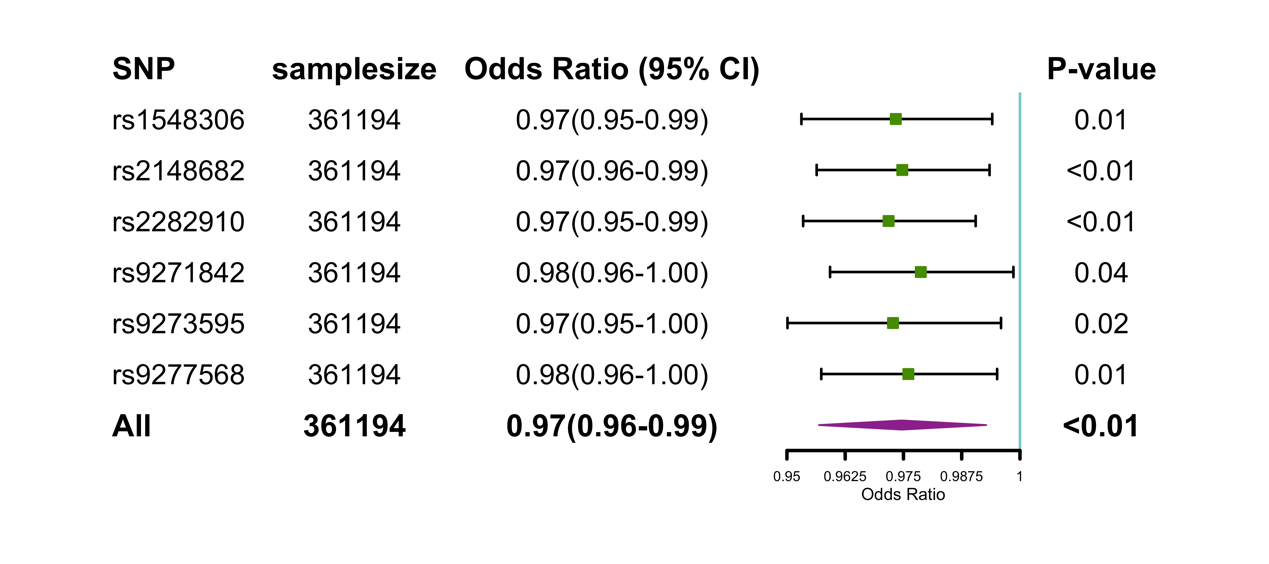


Figure S4. Leave-One-Out Test for Single SNPs Used in the Two-sample Mendelian Randomization Analysis of the Causal Effect of Gluten-Free Diet on Irritable Bowel Syndrome (at *p<1×10^-5^*)

*Abbreviations*: IVW - Inverse variance weighted; CI - confidence intervals.

Table S1. Detail Information of Instrumental SNPs

| **SNP ^1^** | **Chr** | **Position** | **Effect allele** | **Other allele** | **EAF** | **Exposure-GFD** | | | **Outcome-IBS** | | | **Outcome-IBS (validation)** | | |
| --- | --- | --- | --- | --- | --- | --- | --- | --- | --- | --- | --- | --- | --- | --- |
|  |  |  |  |  |  | **Beta** | **SE** | ***P*** | **Beta** | **SE** | ***P*** | **Beta** | **SE** | ***P*** |
| rs1548306 ^*^ | 6 | 32427179 | T | A | 0.67 | -0.0073 | 0.0008 | 1.10E-17 | 0.0002 | 0.0001 | 0.27 | 0.0011 | 0.0003 | 9.40e-04 |
| rs2148682 | 1 | 65869489 | C | T | 0.34 | -0.0038 | 0.0008 | 7.60E-06 | 0.0001 | 0.0001 | 0.47 | 0.0001 | 0.0003 | 0.73 |
| rs2282910 | 7 | 28844824 | T | C | 0.67 | -0.0038 | 0.0009 | 6.40E-06 | -0.0001 | 0.0001 | 0.65 | -0.0001 | 0.0003 | 0.84 |
| rs9271842 ^*^ | 6 | 32594953 | A | C | 0.39 | 0.0058 | 0.0009 | 4.30E-11 | - | - | - | -0.0006 | 0.0003 | 0.07 |
| rs9271847 ^2^ | 6 | 32594993 | G | A | 0.45 | - | - | - | -0.0003 | 0.0001 | 0.04 | - | - | - |
| rs9273595 ^*^ | 6 | 32629091 | G | C | 0.26 | 0.0102 | 0.0009 | 2.10E-28 | -0.0002 | 0.0002 | 0.13 | -0.0005 | 0.0004 | 0.14 |
| rs9277568 | 6 | 33057055 | C | T | 0.31 | 0.0047 | 0.0009 | 6.30E-08 | -0.0002 | 0.0001 | 0.20 | -0.0001 | 0.0003 | 0.77 |

*Abbreviations*: Chr - Chromosome; EAF - Effect allele frequency

^1^ Final selected IVs explained how much the proportion of whole variance of exposure could be referred by variance (R^2^), and R^2^ of GFD was 0.5%.

^2^ As rs9271842 was not included in our outcome database, rs9271847 was used as a proxy.

^*^ rs1548306, rs9271842 and rs9273595 were extracted as instrumental SNPs under a smaller *P* value threshold (*p<5×10^-8^* )

Table S2. Associations between Confounders and Single-nucleotide Polymorphisms via Two-sample Mendelian Randomization (shown with the *P* values)

| SNPs | Education | | circulating leptin levels adjusted for BMI | Average total household income before tax | Age of Smoking Initiation | Alcoholic drinks per week |
| --- | --- | --- | --- | --- | --- | --- |
|  | Age completed full time education | Year ended full time education |  |  |  |  |
| rs1548306 | 0.01 | 0.49 | -^a^ | 0.00 | 0.09 | 0.28 |
| rs2148682 | 0.05 | 0.08 | 0.04 | 0.54 | 0.72 | 0.53 |
| rs2282910 | 0.17 | 0.44 | 0.21 | 0.25 | 0.88 | 0.05 |
| rs9271842 | - | 0.12 | - | 0.08 | - | - |
| rs9273595 | 0.04 | 0.50 | - | 0.20 | 0.25 | 0.76 |
| rs9277568 | 0.84 | 0.01 | 0.08 | 0.68 | - | - |

^a^ The needed information of the SNP is not found in the study used for confounders.
